# Supplementary material for: Evaluation of Cytotoxicity, Release Behavior and Phytopathogens Control by Mancozeb-Loaded Guar Gum Nanoemulsions for Sustainable Agriculture
Source: J Xenobiot. 2023 Jun 5;13(2):270–83. doi: 10.3390/jox13020020 (PMC10305605; doi:10.3390/jox13020020)
Supplement: Supplementary file 1 [file jox-13-00020-s001.zip › jox-2339518-supplementary.pdf]

Supplementary Data:

Evaluation of Cytotoxicity, Release Behavior and Phytopathogens Control by Mancozeb-Loaded Guar Gum Nanoemulsions for Sustainable Agriculture

Ravinder Kumar [1,*], Manju Nehra [2], Dharmender Kumar [3], Baljeet Singh Saharan [4], Prince Chawla [5], Pardeep Kumar Sadh [1], Anju Manuja [6], Joginder Singh Duhan [1,*]

Table S1. Runs to optimize the size of guar gum NEs using response surface methodology.

	**Factor 1**	**Factor 2**	**Response**
	Concentrations		
Run	A:Guar gum %	B:Mancozeb %	Particle size
	w/v	w/v	nm
1	1	0.5	356.7
2	1.5	1.5	394.1
3	0.5	0.5	429.9
4	0.5	1	547
5	1	1	208.1
6	1.5	1.5	205.9
7	0.5	1.5	320.3
8	1.5	0.5	368.3
9	0.5	1.5	559
10	1	1	360.9
11	1	1.5	591.5
12	1	1	460.3
13	1.5	1	246.6
14	1	1	460.4
15	1.5	0.5	356.7
16	1	1	368.3
17	0.5	0.5	460.4

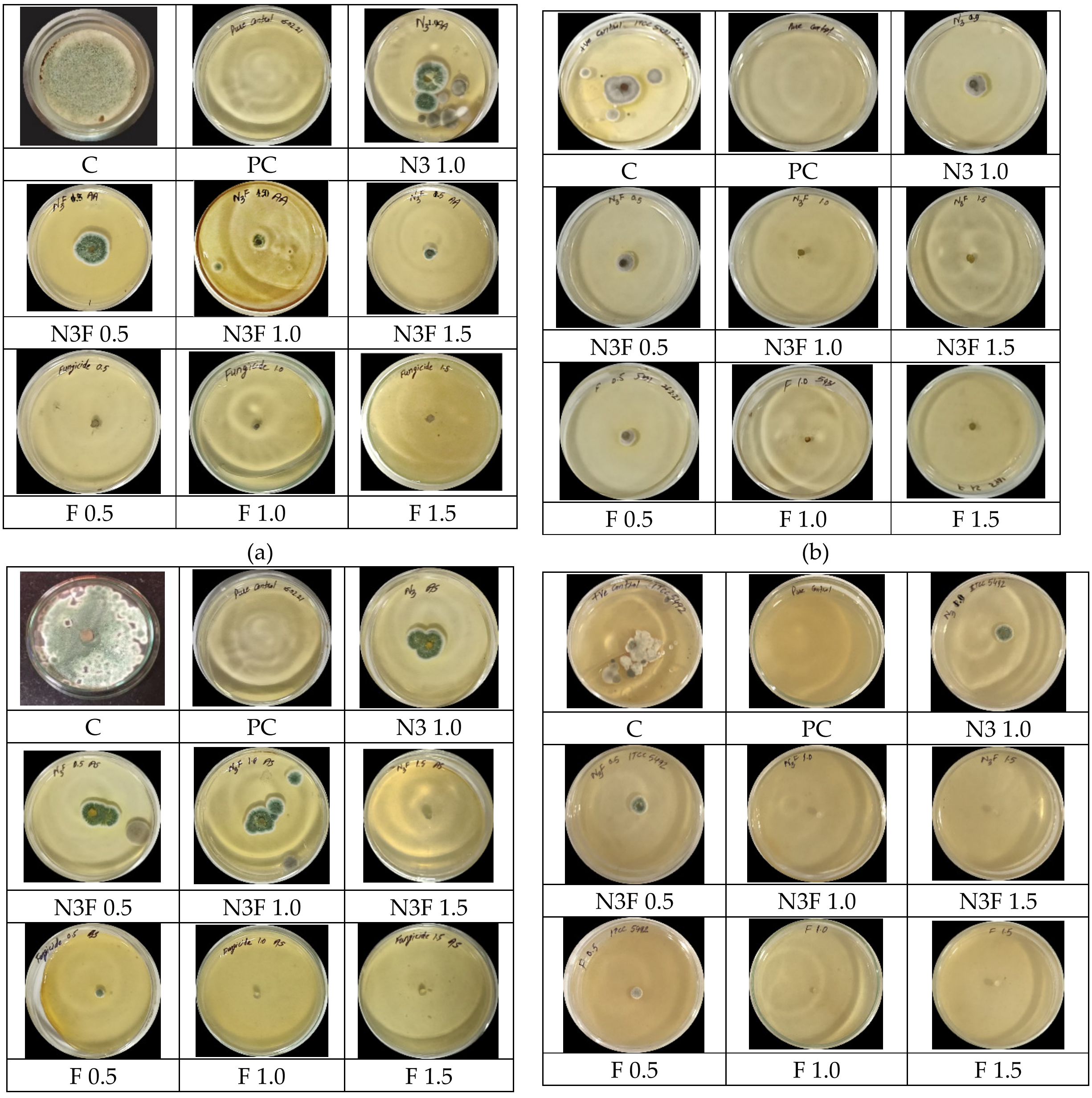

Figure S1. In vitro antifungal ability of blank (N3), and fungicide-loaded (N3F) GG NEs against (a) *A. alternata* (b) *S. lycopersici (c) A. solani (d) S. sclerotiorum*; where: C-control, PC-pure control (media only), N3-blank NEs, N3F-mancozeb (1.0 mg/ml) encapsulated NEs and F- commercial fungicide at 0.5, 1.0 and 1.5 ppm.

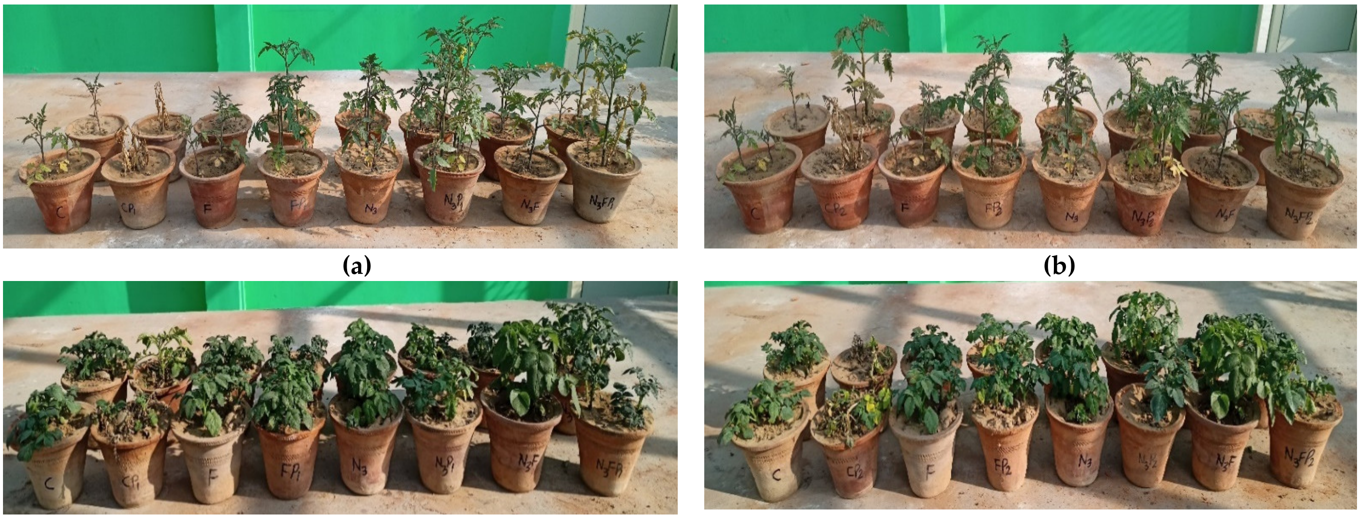

(a) **(b)** **(c)** **(d)**

Figure S2. Disease controlling efficacy of GG NEs on tomato plants (a) early blight (b) leaf spot; on potato plants (c) early blight (d) stem rot; in pot conditions; where C–control (No treatment), CP1-P1, F- fungicide, FP1—fungicide+P1, N3—blank NEs, N3P1—blank NEs+P1, N3F—fungicide-loaded NEs, N3FP1—fungicide loaded NEs+P1, CP2—pathogen P2, FP2-fungicide+P2, N3P2—blank NEs+P2, N3FP2- fungicide-loaded NEs+P2.
